# Trends in microbiological epidemiology of orthopedic infections: a large retrospective study from 2008 to 2021

**DOI:** 10.1186/s12879-023-08471-x

**Published:** 2023-08-31

**Authors:** Boyong Wang, Qiaojie Wang, Musha Hamushan, Jinlong Yu, Feng Jiang, Mingzhang Li, Geyong Guo, Jin Tang, Pei Han, Hao Shen

**Affiliations:** 1 Department of Orthopaedics, Shanghai Sixth People’s Hospital Affiliated to Shanghai Jiao Tong University School of Medicine, Shanghai, 200233 China; 2 Clinical Laboratory, Shanghai Sixth People’s Hospital Affiliated to Shanghai Jiao Tong University School of Medicine, Shanghai, 200233 China

**Keywords:** Orthopedic infections, Microbiological epidemiology, Antimicrobial susceptibilities, Retrospective study, Regional characteristics

## Abstract

**Background:**

This study assessed the distribution characteristics of pathogens isolated from cases of orthopedic infections and focused on the antimicrobial susceptibility of the main pathogens.

**Methods:**

This retrospective study involved patients with orthopedic infection in a tertiary medical center located in Shanghai, China, from 2008 to 2021.Pathogen information and the basic information of patients were identified from clinical microbiology laboratory data and the institutional medical record system.

**Results:**

In total, the pathogen information of 2821 patients were enrolled in the study. *S. aureus* (37.71%) was the main causative pathogen responsible for orthopedic infection. Gender, pathogens distribution and polymicrobial infection rates were significantly different (*P* < 0.05) among patients with different orthopedic infection diseases.The trends in the distribution of pathogens in the total cohort, implant-related infection group (Group A), non-implant-related infection group (Group B), and the sub-group of cases with arthroplasty showed significant linear changes over time. And the polymicrobial infection rates of the total cohort (from 17.17% to 11.00%), Group B(from 24.35% to 14.47%), and the sub-group of cases with internal fixation (from 10.58% to 4.87%) decreased significantly. The antimicrobial susceptibility showed changing trends with time for some main pathogens, especially for *S.aureus* and *Enterobacter spp.*

**Conclusions:**

Our research indicated that the pathogen distribution and antimicrobial susceptibility in orthopedic infections changed over time. And the distribution of pathogens varied significantly among different types of orthopedic infectious diseases. These findings may serve as a reference for prophylaxis and empirical treatment strategies of orthopedic infection.

## Introduction

With the rising quantity of orthopedic surgeries [1], the number of postoperative complications has also increased, among which infection is one of the most serious complications following orthopedic surgeries. The incidence of periprosthetic joint infection (PJI) following primary total hip and total knee replacement is between 0.3% and 1.9%, reaching as high as 10% in revision cases [2, 3]. Moreover, the infection rate following open reduction and internal fixation (ORIF) for fracture is between 1 and 3%, and the infection rate can be as high as 50% for some high-energy and high-risk fractures [4, 5]. Additionally, studies have shown that the success rate of orthopedic infection treatment is only 70–90% [5]. One of the most important aspects of orthopedic infection diagnosis and treatment is the identification of the causative pathogen to provide a guide for decisions on surgical options and local or systemic medications, which will improve the cure rate.

Orthopedic infections are characterized into 2 main categories: implant-related and non-implant-related infections [6]. The little reported research on the differences in the epidemiology and microbiology between implant-related and non-implant related infections, resulting in poor characterization of possible differences in the pathogen distribution in different types of orthopedic infections. The distributions of pathogenic microorganisms responsible for orthopedic infections differ across countries and regions [7–9]. Most studies have been carried out in the USA or Europe, and few data are available based on large contemporary Asian patient cohorts to address these questions. Recent studies suggest that the microorganisms causing orthopedic infections and their antibiotic resistance profiles can change over time, but few studies have presented the trends of orthopedic infection strains over a long period.

Furthermore, epidemiological data on orthopedic infections may be helpful to orthopedic surgeons and infectious disease specialists if the pathogen cannot be identified. Therefore, analyzing the characteristics of pathogen distribution in patients treated with various orthopedic surgeries is important for determining the treatment plan and guiding preventive measures. The aim of this study was to assess the distribution characteristics and trends of pathogens and to compare the differences between different orthopedic diseases with a focus on the antimicrobial susceptibility of the main pathogens in orthopedic infections.

## Methods

### Data collection

This was a single-center retrospective study conducted at a tertiary medical center located in Shanghai, China. The orthopedic surgery department conducts approximately 44,000 orthopedic surgical procedures each year. We retrospectively identified orthopedic infection cases from January 1, 2008, to December 31, 2021, from the electronic database of the clinical microbiology laboratory and the orthopedic medical record system. Data on the time of the procedure, type of orthopedic surgery, basic patient information, infecting pathogens, and antimicrobial susceptibility were collected. We focused our analyses on the five pathogens with the highest proportions. The inclusion criteria included the following: (1) infections occurring only at orthopedic surgical sites or trauma wounds; (2) definitive pathogenic bacterium detection; and (3) clinical manifestations of infection, such as fever, chills, swelling, pus, pain, and elevated skin temperature. The exclusion criteria were as follows: (1) cases lacking relevant medical history information; (2) no clear pathogenic bacterium identification; and (3) infections caused by infection of other sites after orthopedic surgeries (e.g., urinary tract infections, intravenous catheter infections, etc.).

The grouping of patients were dependent on the condition at the time when the pathogens were initially isolated. For patients with recurrent infections and reinfection, only isolates and the clinicial information from the initial positive-culture samples were included. Orthopedic infections were categorized based on the two main sources, implant-related infections (Group A) and non-implant-related infections (Group B). Group A was defined as a surgical site infection occurring after insertion of an implant. Group A was further divided into three subgroups, namely, those with cases with arthroplasty, internal fixation, and external fixation. Group B was defined as an infection that did not involve the implant. Group B was further divided into two subgroups, which included those with musculoskeletal trauma (infection of skin, soft tissue and bone caused by trauma) and those with other non-implant-related infections (Orthopedic related soft tissue infections including diabetic foot infection、necrotizing fasciitis and so on, septic arthritis, hematogenous osteomyelitis, infections following flap/tendon repair, etc.).For patients suffering from a traumatic event, if the pathogens were isolated before the insertion of an implant, the patient was enrolled in musculoskeletal trauma group; If the pathogens were isolated under the situation of patients with an implant, the patient was enrolled in implant-related infections group; If the pathogens were isolated after removing the implant and there were clinical or laboratory signs of infection before and during surgery, the patient was also enrolled in implant-related infections group. While if there were no clinical or laboratory signs of infection before and during surgery, the patient were enrolled in other non-implant-related infections group.

PJI was defined according to the 2018 ICM criteria [10] and fracture-related infection (FRI) containing internal fixation infection and external fixation infection was diagnosed according to the consensus definition for FRI published in 2018 [11] And other SSI were defined according to the CDC criteria [12].

### Microbiological procedures

All isolated strains were obtained from tissues, synovial fluid, pus, and implants. According to the Infectious Diseases Society of America guidelines [13], when the diagnostic standards for orthopedic infections were met, a virulent microorganism (e.g. Staphylococcus aureus) isolated from a single specimen was considered as the causative organism.For low virulent pathogens and/or potential contaminants, such as coagulase-negative staphylococci (CoNS), Corynebacterium spp., and Cutibacterium acnes, at least two culture-positive perioperative and preoperative samples (synovial fluid or pus, three to five intraoperative tissue samples, and prosthetic sonicate fluid) were considered significant for diagnosis. Polymicrobial infection was defined as different pathogens identified simultaneously from samples and we did not consider polymicrobial infection when different CoNS (species or antibiograms) were isolated simultaneously.

The Vitek Compao60 Identification System (BioMérieux Company, France) was used for bacterial identification and antimicrobial susceptibility testing. ATB-FUNGUS fungus identification and susceptibility test strips (BioMérieux, France) were used for *fungus* identification and susceptibility testing. The identification of *M. tuberculosis* was confirmed by TB-DNA detection. TB-DNA was extracted and detected using Diagnostic Kit for Mycobacterium Tuberculosis DNA (PCR-Fluorescence) (Daan Gene, China) and ABI Prism 7500 Real-Time PCR System. The antimicrobial susceptibility of isolates was determined according to the Clinical and Laboratory Standards Institute (CLSI-M100-S20).

### Data analysis

Statistical analyses were performed by using IBM SPSS software (version 23.0; SPSS Inc., IL, USA), and frequency data were analyzed by using the Pearson chi-square test or Fisher’s exact test. Multiple comparison between the groups was performed using Bonferroni method and the different subscript letters indicated significant differences between the groups. The Cochran-Armitage trend test was used to show the linearity of the rate over time, which was performed using JMP, version 16.2.0 (represented by the Z score, a positive score shows an increasing trend, whereas a negative value shows a decreasing trend). A *P* value < 0.05 was considered to indicate statistical significance.

## Results

A total of 2821 patients were included in the study, and 3293 pathogens were identified as causative pathogens. Among them, *S. aureus* was found to be the most common pathogen causing orthopedic infections (Table 1), followed by *Pseudomonas spp.*, *CoNS*, *Enterobacter spp. and Acinetobacter spp..* We categorized the 2821 patients into 2 major groups based on the presence of implant-related (Group A) and non-implant-related infections (Group B). As seen in Table 1, the sex distribution was predominantly male in both Group A and Group B, but the percentage of males in Group A was significantly lower than that in Group B. In terms of pathogen distribution, the proportions of *S. aureus and CoNS* in Group A were significantly higher than those in Group B, while *Pseudomonas spp., Enterobacter spp. and Actinobacteria spp.* were significantly less prevalent in Group A than in Group B. The incidence of polymicrobial infection in Group A was significantly lower than that in Group B.

**Table 1:** Comparison of demographics and pathogens causing infection among patients with or without orthopedic implants

**Variables**	Total, *N* = 2821		***P* value**	**Total**
	Orthopedic implants,* N* = 1441 (51.1%)	No implants, *N* = 1380 (48.9%)		
**Sex [n (%)]**			*P* = 0.008	
Male^*^	994 (69.0)	1014 (73.5)		2008 (71.2)
Female^*^	447 (31.0)	366 (26.5)		813 (28.8)
**Pathogen [n (%)]**	*N* = 1657	*N* = 1636		3293
Staphylococcus aureus^*^	619 (37.4)	458 (28.0)	*P* < 0.001	1077 (32.7)
Pseudomonas^*^	160 (9.7)	224 (13.7)	*P* < 0.001	384 (11.7)
CoNS^*^	266 (16.1)	108 (6.6)	*P* < 0.001	374 (11.4)
Enterobacter^*^	109 (6.6)	154 (9.4)	*P* = 0.003	263 (8.0)
Acinetobacter^*^	92 (5.6)	139 (8.5)	*P* = 0.001	231 (7.0)
Polymicrobial infection rate^*^ **[n (%)]**	146 (10.1)	198 (14.3)	*P* = 0.001	344 (12.2)

‘*’ represents a significant difference (*p* < 0.05) between the orthopedic implant group and no-implant group

*Abbreviations:* *CoNS Coagulase-negative staphylococci*

The results of the subgroup analysis are listed in Table 2. Regarding sex distribution, arthroplasty was the only type of procedure that resulted in a higher rate of orthopedic infection in women. Regarding infectious pathogens, *S. aureus* was significantly more common in cases with internal fixation than in cases with other procedures. *Pseudomonas spp.and Enterobacter spp.*were significantly less common in cases with arthroplasty, whereas *CoNS* was more likely to be associated with arthroplasty; *Actinobacteria spp*. was most commonly observed in patients with musculoskeletal trauma and external fixation.. The musculoskeletal trauma group had the highest rate of polymicrobial infection.

**Table 2:** Comparison of demographics and pathogens causing infection among patients with different orthopedic infections

**Type**	**Implant-related Infections**			**Non-implant related Infections**		
	Internal fixation	Arthroplasty	External fixation	Musculoskeletal trauma	Other non-implant infections	
Number	835	395	211	587	793	**/**
**Sex**						*P* < 0.001
Male (%)*	77.5_a_	45.6_b_	79.1_a_	72.2_a_	74.4_a_	
Female (%)*	22.5_a_	54.4_b_	20.9_a_	27.8_a_	25.6_a_	
**Pathogen (%)**						
Staphylococcus aureus*	49.2_a_	20.4_b_	27.5_b,c_	19.7_b_	35.1_c_	*P* < 0.001
Pseudomonas*	11.3_a_	3.6_b_	15.5_a,c_	16.1_c_	11.7_a,c_	*P* < 0.001
CoNS*	6.3_a_	39.9_b_	4.8_a_	5.0_a_	7.9_a_	*P* < 0.001
Enterobacter*	8.6_a,b_	2.0_c_	8.4_a,b_	12.2_b_	7.0_a_	*P* < 0.001
Acinetobacter*	4.7_a_	3.6_a_	12.4_b_	10.6_b_	6.7_a_	*P* < 0.001
Polymicrobial infection rate (%)*	8.4_a_	12.2_a_	13.3_a,b_	21.0_b_	9.5_a_	*P* < 0.001

‘*’ represents a significant difference (*p* < 0.05) between the subgroups; values with different subscript letters are significantly different (*p* < 0.05)

*Abbreviations:* CoNS, *coagulase-negative staphylococci*

**Fig. 1:**
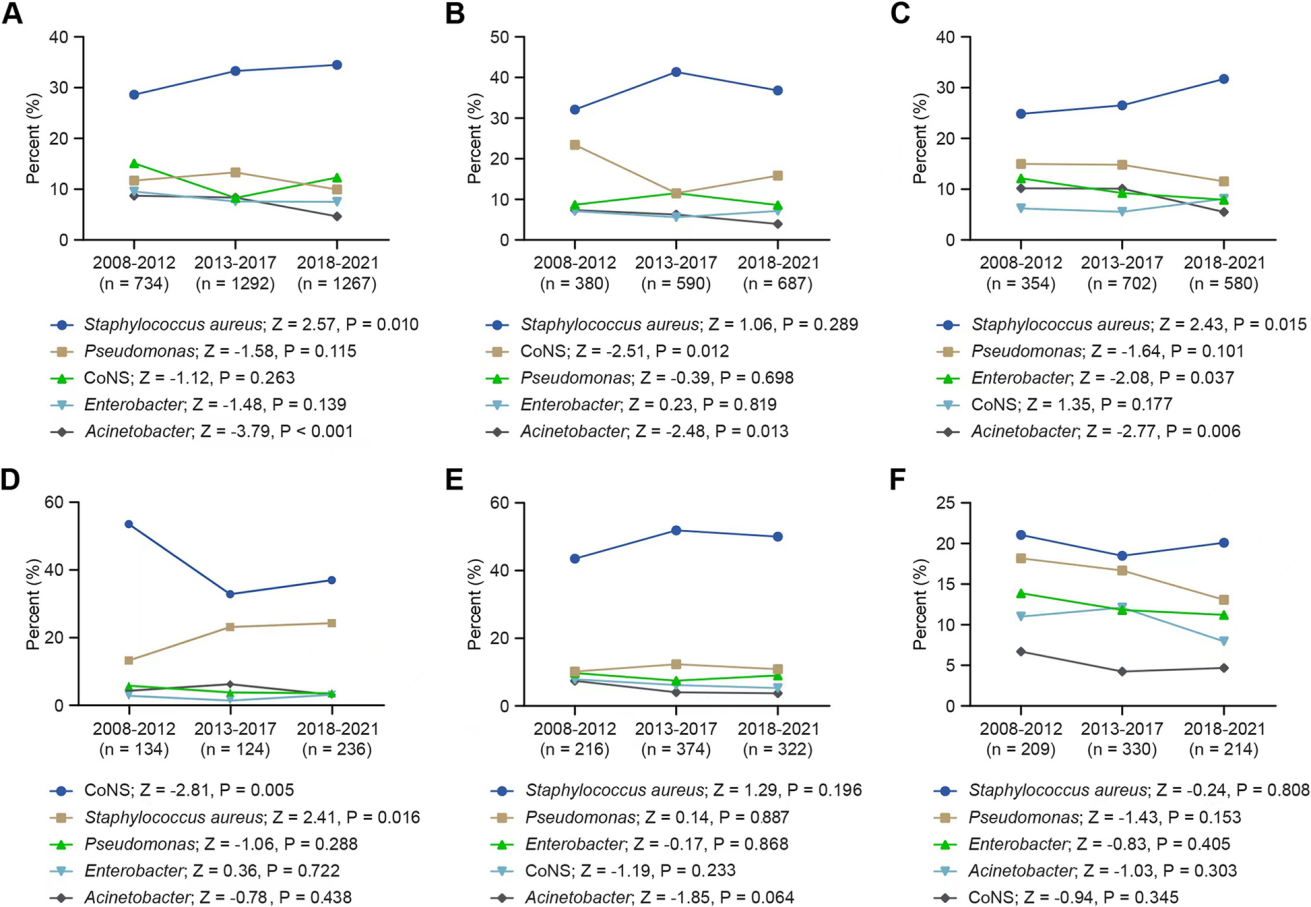
Trends of the proportions of the top 5 pathogens in orthopedic infections from 2008–2011. Trends in the microbial etiology of all cases (**a**), implant-associated cases (**b**), non-implant-associated cases (**c**), arthroplasty-associated cases (**d**), internal fixation-associated cases (**e**) and musculoskeletal trauma-associated cases (**f**) from 2008 to 2021. Z score represents the trends (Z score < 0: downward trend; Z score > 0: upward trend); *P < 0.05 indicates significance. Abbreviations:CoNS,coagulase-negative staphylococci

We then divided the study period of 14 years into 3 intervals to explore the trends in the distribution of the primary pathogens. As shown in Fig. 1a, among all cases, *S. aureus* showed a gradual increase in proportion from 28.61% to 34.49%, whereas *Actinobacteria spp*. continually decreased in proportion from 8.72% to 4.66%, and the other pathogens had a relatively stable distribution with no significant changes. For Group A (Fig. 1b), the proportion (indicated by the proportions in 2008–2011 and 2018–2021, respectively) of *CoNS* (23.42%-15.87%) and *Actinobacteria spp*. (7.37%-3.93%) decreased significantly over the study period, and there were no significant trends for other microorganisms. An increased percentage of *S. aureus* (24.86%-31.72%) was observed in Group B (Fig. 1c), and a significant linear decline in percentage was observed for *Enterobacter spp.* (12.15%-7.93%) and *Actinobacteria spp.* (10.17%-5.52%) over time. Due to the relatively high numbers of patients with arthroplasty, internal fixation and musculoskeletal trauma, we analyzed these three conditions separately. For the arthroplasty group (Fig. 1d), the proportion of *CoNS* (52.99%-36.44%) decreased, and the percentage of *S. aureus (*12.69%-23.73%) increased over time. For the internal fixation and musculoskeletal trauma (Fig. 1e and f), the proportions of pathogens changed steadily, and none of the changes reached statistical significance.

**Fig. 2:**
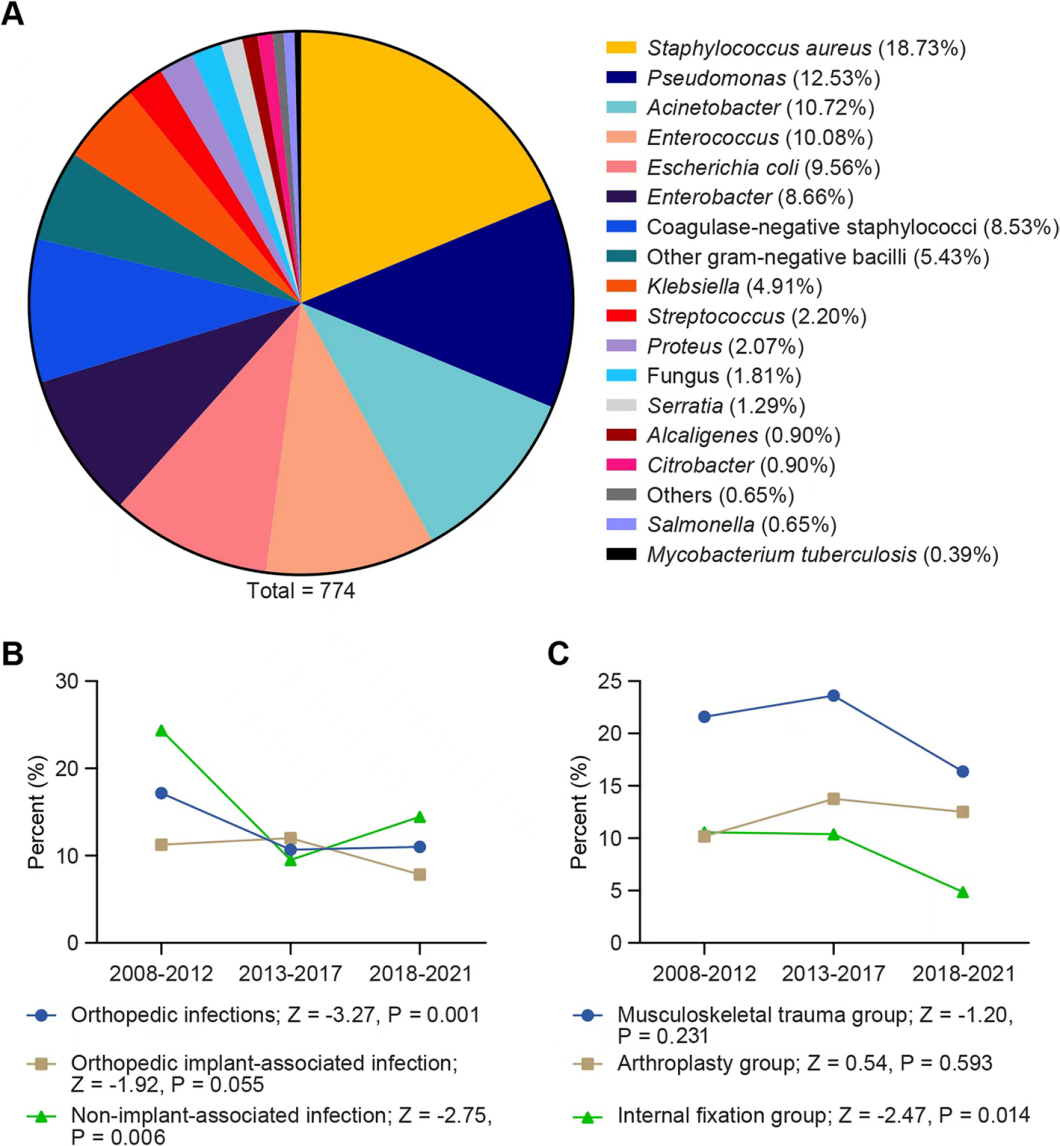
Microbiology of polymicrobial orthopedic infection (**a**). Trends in the polymicrobial infection rates of all cases, implant-associated cases and non-implant- associated cases from 2008 to 2021 (**b**). Trends in the polymicrobial infection rates of arthroplasty-associated cases, internal fixation-associated cases and musculoskeletal trauma-associated cases from 2008 to 2021 (**c**). Z score represents the trends (Z score < 0: downward trend; Z score > 0: upward trend); *P < 0.05 indicates significance

Polymicrobial infection was an important component of orthopedic infections and was identified in 344 patients, accounting for 12.2% of the whole cohort. There were 774 pathogens identified, of which *S. aureus*, *Pseudomonas spp*., *Actinobacteria spp.*, *Enterococcus spp.* and *E. coli* were the five most common pathogens (Fig. 2a). As shown in Fig. 2b, for the total cohort (17.17%-11.00%) and Group B (24.35%-14.47%), the polymicrobial infection rates decreased significantly over the study period. Similarly, the internal fixation group (10.58%-4.87%) showed a significant linear decline in the polymicrobial infection rate. In contrast, the polymicrobial infection rates of the musculoskeletal trauma group and arthroplasty group did not change significantly during the study period (Fig. 2c).

As *S. aureus*, *Pseudomonas spp.*, *CoNS*, *Enterobacter spp.* and *Actinobacteria spp.* were the five most common pathogens, we further focused on the antibiotic susceptibility of these pathogens. The analysis of *S. aureus* showed that 43.9% were *methicillin-resistant S. aureus* (MRSA) and 56.1% were *methicillin-sensitive S. aureus* (MSSA). Similarly, we divided the antimicrobial susceptibility characteristics into 3 stages based on the time of testing (Table 3). The percentage of MRSA decreased significantly over time, from 53.3% in 2008–2012 to 41.2% in 2018–2021. In addition, susceptibility to *gentamicin*, *tetracycline*, *erythromycin*, *clindamycin*, *levofloxacin*, *ciprofloxacin*, *moxifloxacin* and *rifampin* significantly increased among MRSA isolates*.* The proportions of MSSA isolates that were sensitive to *tetracycline* and *erythromycin* also increased significantly, but sensitivity to other antibiotics did not significantly change over time. *Pseudomonas spp.* only showed a significantly decreased susceptibility to *ciprofloxacin* (Table 4)*.* Among the *CoNS* stains (Table 5), 78.3% were *methicillin-resistant coagulase-negative staphylococci* (MRCoNS), and 21.7% were *methicillin-sensitive coagulase-negative staphylococci* (MSCoNS), but the percentages of MRCoNS or MSCoNS did not change significantly over time. Moreover, susceptibility to the tested antibiotics did not significantly change among MRCoNS isolates. However, the proportions of MSCoNS isolates that were sensitive to *levofloxacin* and *ciprofloxacin* decreased significantly. The subsequent analysis of *Enterobacter spp.* (Table 6) showed that the proportions of *Enterobacter spp.* isolates that were sensitive to *tobramycin*, *amikacin* and *gentamicin* increased significantly, and the sensitivity to *ertapenem* decreased significantly over time. However, *Actinobacteria spp.* (Table 7) only showed a significant increase in susceptibility to *amikacin.*

**Table 3:** Temporal trend in percent susceptibility to selected antimicrobials among S. aureus isolates, stratified by methicillin resistance (2008–2021)

**Susceptible by time interval S/S + R (percentage, %)**						
**Antimicrobial agent**	**2008–2012**	**2013–2017**	**2018–2021**	**Z score**	***P* value**	**Overall**
**MRSA [n (%)]**	112 (53.3)	181 (42.1)	180 (41.2)	-2.61	0.009	473 (43.9)
Gentamicin (%)	22/107 (20.6)	130/177 (73.4)	152/180 (84.4)	+ 10.42	< 0.001	304/464 (65.5)
Tetracycline (%)	15/76 (19.7)	119/181 (65.7)	141/180 (78.3)	+ 8.24	< 0.001	275/437(62.9)
Erythromycin (%)	14/110 (12.7)	31/170 (18.2)	77/179 (43.0)	+ 6.06	< 0.001	122/459 (26.6)
Clindamycin (%)	19/109 (17.4)	54/181 (29.8)	70/180 (38.9)	+ 3.83	< 0.001	143/470 (30.4)
Levofloxacin (%)	16/112 (14.3)	123/176 (69.9)	133/180 (73.9)	+ 9.35	< 0.001	272/468 (58.1)
Ciprofloxacin (%)	13/49 (26.5)	105/179 (58.7)	118/180 (65.6)	+ 4.33	< 0.001	236/408 (57.8)
Moxifloxacin (%)	13/55 (23.6)	128/170 (75.3)	140/177 (79.1)	+ 6.51	< 0.001	281/402 (69.9)
Rifampin (%)	63/110 (57.3)	145/181 (80.1)	159/180 (88.3)	+ 5.98	< 0.001	367/471 (77.9)
Vancomycin (%)	109/109 (100)	181/181 (100)	180/180 (100)	/	/	470/470 (100)
Teicoplanin (%)	45/46 (97.8)	113/113 (100)	124/125 (99.2)	+ 0.54	0.586	282/284 (99.3)
**MSSA [n (%)]**	98 (46.7)	249 (57.9)	257 (58.8)	+ 2.61	0.009	604 (56.1)
Gentamicin (%)	84/95 (88.4)	226/249 (90.8)	232/256 (90.6)	+ 0.49	0.621	542/600 (90.3)
Tetracycline (%)	57/78 (73.1)	218/247 (88.3)	231/257 (89.9)	+ 3.26	0.001	506/582 (86.9)
Erythromycin (%)	50/98 (51.0)	133/249 (53.4)	163/255 (63.9)	+ 2.61	0.009	346/602 (57.5)
Clindamycin (%)	71/98 (72.4)	156/247 (63.2)	191/257 (74.3)	+ 1.18	0.239	418/602 (69.4)
Levofloxacin (%)	90/98 (91.8)	235/249 (94.4)	240/257 (93.4)	+ 0.29	0.771	565/604 (93.5)
Ciprofloxacin (%)	59/64 (92.2)	218/240 (90.8)	233/252 (92.5)	+ 0.36	0.723	510/556 (91.7)
Moxifloxacin (%)	55/61 (90.2)	232/236 (98.3)	249/257 (96.9)	+ 1.56	0.118	536/554 (96.8)
Rifampin (%)	95/98 (96.9)	239/246 (97.2)	240/248 (96.8)	-0.15	0.884	574/592 (97.0)
Penicillin (%)	9/98 (9.2)	18/249 (7.2)	30/257 (11.7)	+ 1.16	0.247	57/604 (9.4)
Vancomycin (%)	98/98 (100)	249/249 (100)	256/257 (99.6)	-1.02	0.306	603/604 (99.8)
Teicoplanin (%)	82/82 (100)	170/170 (100)	214/214 (100)	/	/	466/466 (100)

Z score represents the trend (Z score < 0: downward trend; Z score > 0: upward trend); *P* < 0.05 indicates significance

*Abbreviations:* *MRSA* Methicillin-resistant *Staphylococcus aureus*, *MSSA* Methicillin-susceptible *Staphylococcus aureus*, *S* Susceptible, *R* Resistant

**Table 4:** Temporal trend in percent susceptibility to selected antimicrobials among Pseudomonas spp. isolates (2008–2021)

**Susceptible by Time Interval S/S + R (percentage %)**						
**Antimicrobial Agent**	**2008–2012**	**2013–2017**	**2018–2021**	**Z-value**	***P*-value**	**Overall**
**Number**	86	172	126	/	/	
Levofloxacin (%)	62/86 (72.1)	135/166 (81.3)	92/126 (73.0)	-0.10	0.924	289/378 (76.5)
Cefoperazone-Sulbactam (%)	60/84 (71.4)	133/172 (77.3)	84/117 (71.8)	-0.08	0.934	277/373 (74.3)
Ciprofloxacin (%)	67/80 (83.8)	145/172 (84.3)	93/126 (73.8)	-1.99	0.047	305/378 (80.7)
Piperacillin-Tazobactam (%)	75/86 (87.2)	154/172 (89.5)	104/126 (82.5)	-1.16	0.245	333/384 (86.7)
Ceftazidime (%)	71/86 (82.6)	141/172 (82.0)	106/126 (84.1)	+ 0.34	0.730	318/384 (82.8)
Tobramycin (%)	70/86 (81.4)	153/172 (89.0)	111/123 (90.2)	+ 1.82	0.069	334/381 (87.7)
Amikacin (%)	77/86 (89.5)	159/172 (92.4)	114/126 (90.5)	+ 0.13	0.895	350/384 (91.2)
Aztreonam (%)	45/79 (57.0)	100/170 (58.8)	78/126 (61.9)	+ 0.73	0.466	223/375 (59.5)
Gentamicin (%)	68/86 (79.1)	142/172 (82.6)	90/107 (84.1)	+ 0.89	0.371	300/365 (82.2)
Imipenem (%)	75/86 (87.2)	143/172 (83.1)	101/126 (80.2)	-1.34	0.181	319/384 (83.1)
Meropenem (%)	74/86 (86.0)	141/172 (82.0)	100/121 (82.6)	-0.58	0.563	315/379 (83.1)

Z-score represents the trends (Z score < 0: downward trend; Z score > 0:upward trend); *P* < 0.05 indicates significance

*Abbreviations:* *S* Susceptible, *R* Resistant

**Table 5:** Temporal trend in percent susceptibility to selected antimicrobials among CoNS isolates, stratified by methicillin resistance (2008–2021)

**Susceptible by Time Interval S/S + R (percentage %)**						
**Antimicrobial Agent**	**2008–2012**	**2013–2017**	**2018–2021**	**Z-value**	***P*-value**	**Overall**
**MRCoNS [n (%)]**	87 (78.4)	83 (77.6)	123 (78.8)	+ 0.11	0.911	293 (78.3)
Gentamicin (%)	46/87 (52.9)	54/83 (65.1)	75/123 (61.0)	+ 1.07	0.286	175/293 (59.7)
Tetracycline (%)	58/80 (72.5)	62/83 (74.7)	84/122 (68.9)	-0.64	0.520	204/285 (71.6)
Erythromycin (%)	19/87 (21.8)	19/80 (23.8)	33/123 (26.8)	+ 0.84	0.400	71/290 (24.5)
Clindamycin (%)	43/87 (49.4)	39/83 (47.0)	54/123 (43.9)	-0.80	0.425	136/293 (46.4)
Levofloxacin (%)	43/87 (49.4)	42/83 (50.6)	52/121 (43.0)	-0.98	0.326	137/291 (47.1)
Ciprofloxacin (%)	32/71 (45.1)	42/83 (50.6)	50/122 (41.0)	-0.73	0.466	124/276 (44.9)
Rifampin (%)	79/87 (90.8)	74/83 (89.2)	108/123 (87.8)	-0.69	0.493	261/293 (89.1)
Vancomycin (%)	85/86 (98.8)	83/83 (100)	122/123 (99.2)	+ 0.21	0.830	290/292 (99.3)
Teicoplanin (%)	60/63 (95.2)	78/81 (96.3)	111/112 (99.1)	+ 1.59	0.112	249/256 (97.3)
**MSCoNS [n (%)]**	24 (21.6)	24 (22.4)	33 (21.2)	-0.11	0.911	81 (21.7)
Gentamicin (%)	21/24 (87.5)	23/24 (95.8)	31/33 (93.9)	+ 0.85	0.395	75/81 (92.6)
Tetracycline (%)	16/19 (84.2)	21/24 (87.5)	24/26 (92.3)	+ 0.85	0.395	61/69 (88.4)
Erythromycin (%)	13/24 (54.2)	10/24 (41.7)	16/33 (48.5)	-0.36	0.721	39/81 (48.1)
Clindamycin (%)	21/24 (87.5)	17/24 (70.8)	20/33 (60.6)	-2.21	0.027	58/81 (71.6)
Levofloxacin (%)	24/24 (100)	22/24 (91.7)	28/33 (84.8)	-2.01	0.045	74/81 (91.4)
Ciprofloxacin (%)	18/19 (94.7)	21/24 (87.5)	26/26 (100)	+ 0.90	0.366	65/69 (94.2)
Rifampin (%)	22/24 (91.7)	23/24 (95.8)	32/33 (97.0)	+ 0.89	0.373	77/81 (95.1)
Penicillin (%)	10/24 (41.7)	8/24 (33.3)	10/33 (30.3)	-0.87	0.382	28/81 (34.6)
Vancomycin (%)	23/24 (95.8)	24/24 (100)	32/32 (100)	+ 1.33	0.183	79/80 (98.8)
Teicoplanin (%)	22/23 (95.7)	20/20 (100)	33/33 (100)	+ 1.34	0.179	75/76 (98.7)

Z-score represents the trends (Z score < 0: downward trend; Z score > 0:upward trend);*P* < 0.05 indicates significance

*Abbreviations:* *MRCoNS* Methicillin-resistant *coagulase-negative staphylococci*, *MSCoNS* Methicillin-susceptible *coagulase-negative staphylococci,S* Susceptible, *R* Resistant

**Table 6:** Temporal trend in percent susceptibility to selected antimicrobials among Enterobacter spp. isolates (2008–2021)

**Susceptible by Time Interval S/S + R (percentage %)**						
**Antimicrobial Agent**	**2008–2012**	**2013–2017**	**2018–2021**	**Z-value**	***P*-value**	**Overall**
**Number**	70	98	95	/	/	
Levofloxacin (%)	47/70 (67.1)	75/98 (76.5)	69/95 (72.6)	+ 0.68	0.499	191/263 (72.6)
Cefoperazone-Sulbactam (%)	42/59 (71.2)	69/92 (75.0)	63/93 (67.7)	-0.59	0.554	174/244 (71.3)
Ciprofloxacin (%)	48/70 (68.6)	74/98 (75.5)	62/88 (70.5)	+ 0.19	0.850	184/256 (71.9)
Piperacillin /Tazobactam (%)	50/68 (73.5)	81/98 (82.7)	64/95 (67.4)	-1.12	0.262	195/261 (74.7)
Tobramycin (%)	38/66 (57.6)	76/98 (77.6)	74/88 (84.1)	+ 3.65	< 0.001	188/252 (74.6)
Amikacin (%)	58/70 (82.9)	92/95 (96.8)	94/95 (98.9)	+ 4.09	< 0.001	244/260 (93.8)
Aztreonam (%)	30/70 (42.9)	63/98 (64.3)	50/93 (53.8)	+ 1.17	0.241	143/261 (54.8)
Gentamicin (%)	42/70 (60.0)	80/98 (81.6)	80/95 (84.2)	+ 3.49	< 0.001	202/263 (76.8)
Cefepime (%)	50/70 (71.4)	77/98 (78.6)	73/95 (76.8)	+ 0.73	0.464	200/263 (76.0)
Ceftriaxone (%)	22/62 (35.5)	52/98 (53.1)	38/93 (40.9)	+ 0.37	0.709	112/253 (44.3)
Ceftazidime (%)	37/70 (52.9)	65/98 (66.3)	51/95 (53.7)	-0.09	0.931	153/263 (58.2)
Imipenem (%)	70/70 (100)	86/98 (87.8)	87/95 (91.6)	-1.80	0.071	243/263 (92.4)
Meropenem (%)	68/70 (97.1)	95/98 (96.9)	91/95 (95.8)	-0.49	0.622	254/263 (96.6)
Ertapenem (%)	70/70 (100)	91/98 (92.9)	84/95 (88.4)	-2.88	0.004	245/263 (93.2)

Z-score represents the trends (Z score < 0: downward trend; Z score > 0:upward trend); *P* < 0.05 indicates significance

*Abbreviations:* *S* Susceptible, *R* Resistant

**Table 7:** Temporal trend in percent susceptibility to selected antimicrobials among Actinobacteria spp. isolates (2008–2021)

**Susceptible by Time Interval S/S + R (percentage %)**						
**Antimicrobial Agent**	**2008–2012**	**2013–2017**	**2018–2021**	**Z-value**	***P*-value**	**Overall**
**Number**	64	108	59	/	/	231
Levofloxacin (%)	26/56 (46.4)	40/108 (37.0)	28/59 (47.5)	+ 0.14	0.890	94/223 (42.2)
Cefoperazone-Sulbactam (%)	27/60 (45.0)	42/101 (41.6)	33/59 (55.9)	+ 1.19	0.235	102/220 (46.4)
Cefepime (%)	27/64 (42.2)	39/108 (36.1)	24/57 (42.1)	-0.05	0.963	90/229 (39.3)
Ciprofloxacin (%)	27/64 (42.2)	40/108 (37.0)	9/20 (45.0)	-0.14	0.890	76/192 (39.6)
Ceftazidime (%)	28/64 (43.8)	41/107 (38.3)	29/59 (49.2)	+ 0.57	0.568	98/230 (42.6)
Tobramycin (%)	29/55 (52.7)	46/108 (42.6)	11/20 (55.0)	-0.38	0.707	86/183 (47.0)
Amikacin (%)	31/64 (48.4)	58/104 (55.8)	45/59 (76.3)	+ 3.11	0.002	134/227 (59.0)
Ampicillin-Sulbactam (%)	28/64 (43.8)	34/83 (41.0)	20/46 (43.5)	-0.07	0.945	82/193 (42.5)
Gentamicin (%)	29/64 (45.3)	41/107 (38.3)	22/46 (47.8)	+ 0.12	0.902	92/217 (42.4)
Imipenem (%)	23/40 (57.5)	33/73 (45.2)	30/57 (52.6)	-0.33	0.743	86/170 (50.6)
Meropenem (%)	33/60 (55.0)	45/92 (48.9)	27/49 (55.1)	-0.05	0.961	105/201 (52.2)
Ertapenem (%)	9/51 (17.6)	21/91 (23.1)	4/15 (26.7)	+ 0.89	0.372	34/157 (21.7)

Z-score represents the trends (Z score < 0: downward trend; Z score > 0:upward trend); *P* < 0.05 indicates significance

*Abbreviations:* *S* Susceptible, *R* Resistant

## Discussion

This single-center retrospective study provides information about the changes in the prevalence of pathogens and the distribution of pathogens among different types of orthopedic infections. This study also highlights the antimicrobial susceptibility characteristics of the main pathogens in orthopedic infections over the past 14 years and thus provides a basis for refining the choices of empirical antimicrobial strategies.

In this study, *S. aureus* was the major pathogen causing orthopedic infections. It is well established that *S. aureus* is the most common pathogen in many orthopedic infections, accounting for 31.7%-43.6% [6, 14, 15]. We then analyzed the distribution of pathogens in different orthopedic infections. The diversity of implants, local microenvironment, and pathogen adaptation are responsible for the different proportions of pathogens in different orthopedic infections. Moreover, the local microenvironment may vary with the anatomical sites of surgery and the tissues involved, the physical and chemical characteristics of the implanted biomaterial, and the type of individual tissue response [16]. In the comparisons between Group A and Group B, we found that *S. aureus and CoNS* were significantly more likely to be associated with orthopedic implants. Implant-associated infections can lead to biofilm formation, and the results may be due to the fact that *S. aureus and CoNS* have multiple mechanisms for attachment and biofilm formation that contribute to their association with implant infections [17, 18]. Among non-implant-related infections, *gram-negative bacilli* (GNB) were predominant, which may be related to the fact that most of these patients with these infections suffered an open fracture after direct trauma and/or infection caused by soft tissue injury [19]. It has been reported that a high incidence of GNB, mainly *Enterobacteriaceae*, is common in developing countries [20, 21]. These results are consistent with the findings in studies by Montanaro et al. [6] and Arciola et al. [16]., who reported that the frequency of *staphylococci* appeared higher in medical device (MDS) infections and that GNB were more frequent among non-MDS-associated infections.

After comparing the differences between the two major groups, we further analyzed the subgroups. Regarding sex, men were engaged in more manual labor and physical activity than women, while the incidence of osteoarthritis was higher in women, which may account for orthopedic infections being predominantly identified in males except for arthroplasty-associated infections. Arthroplasty-associated infections may be an influential factor in the lower proportion of males with implant-associated infections. In this study, we found that *S. aureus* was the dominant pathogen in cases with internal fixation-associated infection, whereas *CoNS* was the major pathogen in PJI. This discrepancy is likely due to the differences in the interstitial milieu of implants. However, Rosteius et al. [7] analyzed 937 patients with hip and knee PJI between 2003 and 2011 and found that *S. aureus* was the predominant pathogenic type. Moreover, Triffault-Fillit et al. [22] counted the pathogen differences in 567 PJI cases according to the time of occurrence from prosthesis implantation and revealed the following: in early/delayed infections, *S. aureus* was the main pathogen; in late acute infections, *Streptococcus spp.* was the most common, and *CoNS* were the most frequent pathogens only in the late chronic types of infection. Different regions, periods, races and infection types may result in different pathogen prevalences. Furthermore, lower proportions of *Pseudomonas spp.*, *Enterobacter spp. and Acinetobacter spp*. in the arthroplasty group were observed in our study. The characteristics were consistent with the results reported by Hu et al. [23] and Wang et al. [8]. PJIs caused by GNB were mostly acute hematogenous infections that originated from urinary tract infections [22], and the low incidence of the mechanism of acquisition could explain these results. Exposure to the environment is a common feature of external fixation and musculoskeletal trauma, which may be responsible for the larger proportions of GNB [19]*.* Overall, there were significant differences in the distribution of pathogens between different types of orthopedic infections. These differences can be used to guide the empirical use of antibiotics if the pathogens are unknown.

Regarding the trend of the pathogen distribution over the past 14 years, the proportion of *S. aureus* in Group A did not change significantly, whereas that in Group B showed a significant increasing trend. Therefore, the significantly increasing proportion of *S. aureus* in the total cohort was mainly influenced by the change in Group B. The proportion of *Actinobacteria spp*. and *Enterobacter spp.* decreased in the two major groups; these bacteria are the main hospital-associated pathogens. Strict aseptic operation, hand hygiene, and cleaning and disinfection procedures have contributed to this change. Moreover, we found a decreasing trend of *CoNS* and an increasing trend of *S. aureus* in the arthroplasty group over time, which could explain the decreasing proportion of *CoNS* in Group A. However, Wang et al. [8] observed a decreasing percentage of *S. aureus* and a rising percentage of *CoNS* in a retrospective study of 10,768 patients who received primary total knee arthroplasty (TKA) from 2002 to 2014. In a retrospective study of 2524 patients with PJI from 19 hospitals between 2003 and 2012 [9], it was found that the proportion of *S. aureus* and *CoNS* was consistently stable and did not change significantly. The differences may be caused by the different antimicrobial usage in different regions, there is also heterogeneity in the prevalence of pathogens according to geographical areas.

Polymicrobial infection is an important constituent of orthopedic infection, which cannot be ignored. The management of patients with polymicrobial orthopedic infections requires the administration of a broad-spectrum antibiotic or often multiple antibiotics to ensure adequate coverage against the infecting organisms. The treatment of these patients may be associated with a higher failure rate, mortality, and treatment costs than those of patients with monomicrobial infections [24]. Thus, we then analyzed mixed orthopedic infections, and *S. aureus* remained the most common pathogen. However, the polymicrobial infection group had a higher proportion of *GNB* (57.6% vs. 44.4%) and *Enterococcus spp*. (10.1% vs. 5.2%). Peel et al. [25] and Tan et al. [24] found a similar association between polymicrobial infection and infection with *GNB* and *Enterococcus spp*. Musculoskeletal trauma was the main component in Group B, and this mechanism results in direct contact with the external environment. Musculoskeletal trauma wounds can be easily contaminated by the external environment, which may account for the high polymicrobial infection rates of the no-implant group and musculoskeletal trauma group [26, 27]. Previous studies asserted that polymicrobial infection usually occurs in the early stage and that dehiscence after surgery is more common in polymicrobial than in monomicrobial infections [28]. Regarding the trend of the polymicrobial infection rate, the total cohort, Group B,and subgroup with internal fixation demonstrated a decreasing trend, which may be due to improved postoperative wound care and antibiotic prophylaxis.

In this retrospective study, we focused on the antimicrobial susceptibility of the main pathogens in orthopedic infections. We found that the percentage of infections caused by MRSA decreased over time. In a study of 191,460 *S. aureus* isolates collected at 427 centers [29], the overall MRSA rates declined between 2005 and 2016, which is consistent with the results of the current study. A decreasing trend was observed in the overall prevalence of MRSA infection in China [30], and a marked decrease in the prevalence of MRSA among Chinese hospitals within recent years has already been reported by the China Antimicrobial Surveillance Network (CHINET) [31]. Moreover, we found that the susceptibility to some antibiotics of MRSA increased over time. Dickstein et al. [32] and Diekema et al. [29] also found that the susceptibility of MRSA isolates to some older antibiotics was increasing, a possible result of the epidemic spread of MRSA clones that are more susceptible to these agents, whereas antibiotic susceptibility was found to be stable in a retrospective study by Zhang et al. [33] of 61,045 *S. aureus* isolates from 226 centers between 2012 and 2017. Ongoing surveillance and further research are required to detect future waves of MRSA epidemics and resistance in orthopedic infections. The analysis of *S. aureus* revealed that the proportion of MSSA was slightly higher than that of MRSA. However, among *CoNS*, the proportion of MRCoNS was significantly higher than that of MSCoNS. Tsai et al. [34] also reported these differences, which may be due to the difference in the adaptive capability to changing environmental conditions between *staphylococci*. Regarding *CoNS*, *Pseudomonas spp.* and *Actinobacteria spp*., the susceptibility of these isolates to some common antibiotics was found to be stable over time. However, *Enterobacter spp.* showed a decreasing trend of susceptibility to carbapenem antibiotics. This finding is consistent with the finding that c*arbapenem-resistant Enterobacteriaceae* (CRE) infection is highly endemic in China [35]. Although CRE is not the major pathogen in orthopedic infections, this increasing trend still deserves increased concern.

This study has several limitations. First, we assessed the microbiological epidemiology of only culture-positive orthopedic infections. Culture-negative orthopedic infections are also an important part, and it has been reported that culture-negative orthopedic infections are caused mostly by fungi and *Mycobacterium tuberculosis *[36], which may have led to their low proportions of these two pathogens in our study. In addition, the data represent the experience from a single center, which exposes the results to a risk of local epidemiologic bias and thus may not be indicative of the experience of others.

## Conclusion

In conclusion, this is the largest study reporting information on the microbial etiology of orthopedic infections in Asian population. The proportions of pathogens varied dramatically among different orthopedic infectious diseases. Moreover, the main pathogens, particularly *S. aureus* and *Enterobacter spp.*, showed variations in antimicrobial susceptibility over time. These results can provide a basis for formulating effective preventive measures and treatment plans and reduce the burden of treatment associated with orthopedic infections. Nevertheless, large multicenter studies with larger time spans are needed to validate the findings.

## Data Availability

All data generated or analysed during this study are included in this published article.
